# Things That Go Bump in the Literature: An Environmental Appraisal of “Haunted Houses”

**DOI:** 10.3389/fpsyg.2020.01328

**Published:** 2020-06-12

**Authors:** Neil Dagnall, Kenneth G. Drinkwater, Ciarán O’Keeffe, Annalisa Ventola, Brian Laythe, Michael A. Jawer, Brandon Massullo, Giovanni B. Caputo, James Houran

**Affiliations:** ^1^Department of Psychology, Manchester Campus, Manchester Metropolitan University, Manchester, United Kingdom; ^2^School of Human & Social Sciences, Buckinghamshire New University, Buckinghamshire, United Kingdom; ^3^Parapsychological Association, Columbus, OH, United States; ^4^Institute for the Study of Religious and Anomalous Experience, Jeffersonville, IN, United States; ^5^Independent Researcher, Medina, OH, United States; ^6^Emotion Gateway Research Center, Vienna, VA, United States; ^7^DISTUM, University of Urbino, Urbino, Italy; ^8^Laboratory for Statistics and Computation, ISLA—Instituto Politécnico de Gestão e Tecnologia, Vila Nova de Gaia, Portugal; ^9^Integrated Knowledge Systems, Dallas, TX, United States

**Keywords:** ghost, haunt, anomalous experiences, environmental sensitivities, phenomenology

## Abstract

This paper contains a narrative overview of the past 20-years of environmental research on anomalous experiences attributed to “haunted house.” This exercise served as a much-needed update to an anthology of noteworthy overviews on ghosts, haunts, and poltergeists (Houran and Lange, 2001b). We also considered whether new studies had incorporated certain recommendations made in this anthology. Our search revealed a relative paucity of studies (*n* = 66) on environmental factors that ostensibly stimulate haunt-type experiences. This literature was diverse and often lacked methodological consistency and adherence to the prior suggestions. However, critical consideration of the content revealed a recurring focus on six ambient variables: embedded (static) cues, lighting levels, air quality, temperature, infrasound, and electromagnetic fields. Their relation to the onset or structure of witness reports showed mostly null, though sometimes inconsistent or weak outcomes. However, such research as related to haunts is arguably in its infancy and new designs are needed to account better for environmental and architectural phenomenology. Future studies should therefore address four areas: (i) more consistent and precise measurements of discrete ambient variables; (ii) the potential role of “Gestalt influences” that involve holistic environment-person interactions; (iii) individual differences in attentional or perceptual sensitivities of percipients to environmental variables; and (iv) the role of attitudinal and normative influences in the interpretation of environmental stimuli. Focused scrutiny on these issues should clarify the explanatory power of evolutionary-environmental models for these and related anomalous experiences.

## Introduction

It might sound like an amusing or curious claim, but “haunted houses” could be among the oldest problems in environmental psychology, i.e., the scientific study of the transactions and interrelationships between people and their surroundings (Bell et al., 2001; Devlin, 2018). In this context, anthropologists note that haunt experiences have been important aspects of shamanism in both early and contemporary societies (Hunter, 2018; McClenon, 2004; Winkelman, 2004). For instance, “shaking tent” rituals involved a special cylindrical lodge or tent to contact “spirits” for guidance on hunting, healing, and even locating missing persons. And as the name of the ritual suggests, the tent is supposed to tremble mysteriously during the ceremony.

Moreman (2018, p. 29) credited “the earliest haunted house story in Greek or Roman literature” to a 2,000-year-old play by Plautus entitled “Mostellaria” (ca 200-194 B.C.E). This fictitious story reveals Roman beliefs about such phenomena (Felton, 1999), which seemingly align well with modern presumptions (Goldstein et al., 2007; Hunter, 2018; Massullo, 2019). Haunt-type experiences can also be found across many different societies and eras (Carrington and Fodor, 1951; Owen, 1964; Roll, 1977; Gauld and Cornell, 1979/2017; Finucane, 1996; Tuczay, 2004). Still, it is important to note that the sociocultural milieu influences the interpretation of these anomalous episodes and the ways people cope with them (for discussions, see Houran, 2004; Houran and Lange, 2001b).

### Haunted Houses as Social Facts…and Possibly More

The term “haunted house” references two types of ostensibly anomalous episodes, which we will examine from a phenomenological perspective (Lange and Houran, 2001a; Houran et al., 2019a, b). First, “poltergeist disturbances” can be described as clusters of unusual experiences (e.g., apparitions, sensed presences, hearing voices, and unusual somatic or emotional manifestations) and physical events (e.g., objects appearing to move by themselves, malfunctioning electrical or mechanical equipment, and inexplicable percussive sounds such as raps or knocks), which focus around certain people (Roll, 1977; Ventola et al., 2019).

Similar anomalies that persist at specific locations are said to constitute a second classification, “haunts” or “hauntings” (Gauld and Cornell, 1979/2017; Roll and Persinger, 2001). However, a firm distinction between these two types of occurrences is tenuous due to their overlapping characteristics (Dixon et al., 2018; Houran et al., 2019b; Ventola et al., 2019) and shared set of psychological and physical anomalies that conform to a unidimensional and probabilistic (Rasch) hierarchy (Houran and Lange, 2001a; Houran et al., 2002b, 2019a). Thus, a common source or set of mechanisms might underlie both kinds of episodes.

Skeptical readers should not trivialize these anomalous experiences because they can affect several facets of people’s lives. Fundamentally, belief in ghosts informs an individual’s religio-cultural worldview (Dyne, 2010; Eaton, 2015, 2019; Hill et al., 2018). One journalist detailed an interesting and practical example of this during the COVID-19 pandemic (Purwanto, 2020). These beliefs likewise have implications for social identity theories of social rank, self esteem, and the belief systems that individuals hold for explanatory meaning in their lives (Tajfel and Turner, 1979; Dagnall et al., 2015b; Hill et al., 2019). Reports of ghosts and haunts may also reinforce or contextualize the anxieties of individuals who are already fearful of the paranormal (Lange and Houran, 1999; de Oliveira-Souza, 2018).

The influence of haunt-type episodes is clearly widespread. Gallup polls from 1990, 2001, and 2005 showed that a substantial proportion of respondents believe in ghosts and hauntings (Dagnall et al., 2015a, 2016). The 2005 Gallup poll found that 37% of those surveyed believed that houses could be haunted, with 32% stating that the spirits of deceased people could return to certain places or situations (Moore, 2005). Other surveys have reported similar or even higher figures (e.g., Pew Research Center, 2009; Live Science, 2011; Lipka, 2015). Particularly, Chapman University (2018) Survey of American Fears documented from 2016 to 2018 an 11% rise in respondents who “agree” or “strongly agree” with the statement: “Places can be haunted by spirits.” The percentage for 2018 (the last time the survey was conducted) was an astounding 58%. Likewise, a YouGov (2019) study reported that 45% of Americans polled believe that ghosts “definitely” or “probably” exist.

Such beliefs can also spur various social consequences. For instance, the ghostly reputations of certain places have sometimes provoked real estate lawsuits concerning undisclosed “stigmatized properties” (Murray, 2017). In fact, houses rumored to be haunted often suffer significant value diminution, and this is especially true in countries such as Taiwan and Hong Kong (China) where haunts have strong cultural associations with bad luck, vengeful ghosts, and ancestral spirit anger (Emmons, 1982; Chu, 2016; Bhattacharya et al., 2017). On the other hand, “haunted” buildings or sites in Western culture are often promoted favorably as tourist destinations (Hanks, 2015; Houran et al., 2020). Sales of these places can bring high prices if a paranormal reputation is regarded as a benefit by buyers (Behar, 2017).

Hill et al. (2018) further discussed how entire industries have developed around “legend-tripping,” i.e., deliberately visiting spooky locations to seek paranormal experiences (cf. Bird, 2002; Holloway, 2010), as well as virtual excursions pursued via live-streamed videos (Kinsella, 2011). Folklorists might regard such pursuits as examples of “ostension,” which is showing or acting out a legend’s narrative in real life (Manning, 2018). These trends relate to broader issues, such as Maddern and Adey (2008) concept of spectro-geographies. Edensor (2008) echoed this perspective in asserting that “ghosts ‘are a ubiquitous aspect of the phenomenology of place,’ ‘ineffable and quasi-mystical’ dimensions which emerge in encounters with the material, the mediated, the sensual and the affectual” (p. 331). As such, spectral themes frequently appear in the popular media and academic literature. All of this serves to promote ghosts and haunted houses as pervasive cultural narratives (Edwards, 2005; Goldstein et al., 2007; Booker, 2009; Lecouteux, 2012; Bader, 2017), which can become highly engaging memes (Hill et al., 2018, 2019; Drinkwater et al., 2019).

Overall, haunted houses exist as psychological, cultural, economic, and legal realities — with a strong and engaging “brand personality” akin to popular consumer products (Annett et al., 2016; Hill et al., 2018, 2019; Houran et al., 2020). Although witness accounts are often explained away as instances of gullibility, overactive imaginations, or outright fraud (Nickell, 2001, 2012; Ashford, 2017), researchers have occasionally documented the presence of independent environmental or physical mechanisms in spontaneous cases (e.g., Persinger and Koren, 2001b; Vinokur, 2005, 2016; Nickell, 2008; Colvin, 2010; Laythe and Houran, 2019). The ontological status of these anomalous episodes beyond that of social facts thus remains a legitimate question. Consistent with this perspective, our paper evaluates the broad explanatory power of environmental psychology for haunt-type experiences.

### The Present Review

This paper extends Houran and Lange (2001b) noteworthy series of peer-reviewed and multidisciplinary research overviews on “ghostly episodes” (i.e., ghosts, haunts, and poltergeists) by focusing on subsequent academic work related to environmental factors. This update is needed as researchers have published an array of relevant articles over the past two decades. To be sure, it is good practice to perform a periodic synthesis of subject knowledge accruing from the rapid rate of publications (Ferrari, 2015), as well as to collate and share key research on a topic (Bolderston, 2008).

We also checked whether methodological recommendations made by several authors in the anthology (Houran and Lange, 2001b) benefitted subsequent research. These recommendations called for (i) more holistic studies that simultaneously consider and measure a range of potential physical factors in haunted environments (e.g., Radin, 2001; Roll and Persinger, 2001), (ii) the use of proper control conditions to understand the naturally occurring presence (or expected fluctuations) of physical variables as compared to haunted locations (e.g., Persinger and Koren, 2001b), and (iii) extensive or competitive hypothesis testing involving cooperation between skeptics and parapsychologists (e.g., Schmeidler, 2001). Critical consideration of the effectiveness of these refinements is vital to advance a scientific understanding of haunt-type experiences (for a discussion, see Houran, 2017).

Consequently, we conducted a non-systematic or narrative overview to identify, assess, and synthesize the relevant literature (Ferrari, 2015). This was done in preference to a systematic review for several reasons (cf. Green et al., 2006; Gregory and Denniss, 2018). Our goal was to identify important research rather than all articles produced within the specified period. Hence, our analysis focused on significant indicative research, intending neither to be exhaustive nor definitive. This methodology is potentially open to bias, but the use of specific research terms ensured that the studies assessed were thematically congruent with the topic of environmental factors in haunt-type experiences. We explicitly provide an overview of the relevant research area and evaluate the current status of the topic but note that a detailed critique of each identified study is not necessarily a property of this approach (cf. Helewa and Walker, 2000; Green et al., 2006). A systematic review, in contrast, locates all relevant published and unpublished studies with the intention of assessing publication impact and identifying bias. A systematic review also tests specific hypotheses or examines the impact of specific populations, outcomes, etc. (Gregory and Denniss, 2018).

Commensurate with our stated aims, meta-analysis was deemed not appropriate. Meta-analysis is a quantitative, formal study design used to systematically assess the results of previous research in order to derive conclusions about that literature (see e.g., Forero et al., 2019). However, several issues argue against the use of meta-analysis here. Recent research shows that at least five compatible studies are needed to sufficiently overcome between study error variance (Jackson and Turner, 2016). On this point, crucial disparities exist among the research methods and contexts in the literature that we sourced. These differences are substantial; they include purely field versus laboratory physical readings and, in some instances, a lack of either.

Moreover, in many instances the physical aspect of what was being assessed (e.g., EMF variation, frequency, or magnitude, and sometimes infrasound) are not convertible to a standard metric given the information provided by the studies. A meta-analysis, therefore, could produce inaccurate results and interpretations. Also, despite their popularity, meta-analytic techniques are not without criticism (Thompson and Pocock, 1991; Sharpe, 1997; Houran et al., 2018). Regardless of the method employed, a sound review is characterized by rigorous evaluation and critical analysis of relevant academic work (Bolderston, 2008).

## Method

We reviewed conceptual and empirical research on “ghostly episodes” from environmental perspectives that was published primarily since the evaluations in Houran and Lange (2001b). We targeted studies using eighteen keywords or phrases frequently used in research examining haunt-type experiences: anomalous experience, apparition, demon, ego-alien intrusions, encounter experiences, ghost, ghost-hunting, haunt, haunting, metachoric experience, paranormal belief, paranormal experience, poltergeist, possession, séance, sensed presence, sitter-group work, and spirit.

Our search was limited to publications written in English. The procedure covered electronic search engines and repositories (i.e., Google Scholar, PsycINFO, and ResearchGate), and examination of titles, abstracts, reference lists, and publications. Finally, we included studies cited in the sourced works. This process yielded 66 articles. Of these, 55% (*n* = 36) appeared in parapsychology journals or niche sources versus 45% (*n* = 30) in mainstream journals or sources. The distribution appears reasonably balanced from an ideological standpoint, but this literature set averages just three articles per year. This implies slow and limited progress in examining the role of environmental factors in haunt-type episodes.

## Results

Six environmental variables emerged from our qualitative inspection of the identified literature: (i) embedded (static) cues; (ii) lighting levels; (iii) air quality; (iv) temperature; (v) infrasound; and (vi) electromagnetic fields. Study authors often characterized these factors as either conscious or unconscious stimulants of anomalous experiences. Conscious stimulants are variables that can overtly capture attention and be interpreted as ghostly. Unconscious (or non-conscious) stimulants refer to elements that are unwittingly or passively sensed and can stimulate unusual or anomalous perceptions. Note that some variables might act as either type of stimulant.

### Embedded (Physical or Static) Cues in the Environment

Content or thematic analyses of haunt narratives have suggested that the perceptual details of percipients’ experiences are often congruent with contextual variables attending the situation or location (Harte, 2000; Houran, 2000). These “context effects” include tangible embedded cues in the physical environment, such as the mysterious aroma of lilacs in a room with a prominent violet hue or the mysterious sound of waltz music in an empty ballroom. Unfortunately, few ecologically valid studies have empirically tested the premise that haunt experiences might involve such cues.

There are two notable exceptions. First, Houran (2002) examined the real-time anomalous experiences of research participants in tandem with salient environmental and aesthetic characteristics of haunted vs non-haunted rooms in a historic mansion with a quiet reputation for paranormal activity. For instance, certain locations were associated with reports of a sensed presence. These accounts correlated with rooms containing the most artwork (i.e., portrait paintings that conceivably created a sense that participants were literally being watched). Nonetheless, analysis found no statistically significant differences between the haunted and non-haunted areas based on the number of pieces of artwork (specifically paintings and sculptures), or the ambient temperature, humidity, or the number of air vents in each room. Consequently, there was no evidence that these environmental features acted as contextual variables to stimulate or shape participants’ experiences.

Terhune et al. (2007) improved on this basic procedure in their field investigation of a reputed haunt (an unpublicized private residence) in comparison to a nearby control house. These researchers measured physical cues such as windows, mirrors, and the quantity and type of artwork (with and without human forms) using a research design that encompassed (i) potential differences between the target and control houses; and (ii) potential differences within haunted and non-haunted areas of the target house. These physical cues were also examined in relation to the presence of apparent photographic anomalies obtained across different film media during the study and rated by independent judges.

Similar to Houran (2002), no statistically significant effects (*p* < 0.05) were found for the environmental variables. However, there was a suggestive trend (*p* < 0.07, two-tailed) for the control house (*M* = 3.57, *SD* = 3.10) to contain more mirrors than the target house (*M* = 1.00, *SD* = 1.41). This finding might seem surprising and counterintuitive, since mirrors and reflective surfaces in general are associated with anomalous experiences (Caputo, 2010a,b; Caputo, 2013; Caputo, 2015; Caputo, 2016; Caputo, 2017; Caputo, 2019; Caputo et al., 2012). This correlation offers several interpretations. Mirrors might, for example, serve as embedded (physical) cues that reinforce expectancy or suggestion effects. However, this possibility must be balanced against the evidence indicating that reflective surfaces can directly stimulate perceptual aberrations, independently of suggestion (Caputo, 2010b, in press).

Indeed, unusual or anomalous perceptions (in different sensory modalities) predictably and systematically manifest when even healthy (i.e., non-clinical) individuals are directed to stare intently into a mirror, darkened space, or another person’s face over a period of time and under low illumination (Caputo, 2019). Psychomanteum, mirror-gazing, and eye-gazing protocols that are used to study these perceptual phenomena constitute a fascinating niche within consciousness studies and can aid model-building or theory-formation of haunt-type experiences (cf. Radin, 2001). Caputo (2019) proposed three distinct clusters (or factors) of anomalous experience that derive from different brain circuits stimulated during such facilitated sessions. He validated his idea with a questionnaire study that assessed the strength and frequency of a large list of apparitional and anomalous phenomena. This list generally aligned with Baker (2002) definition of apparitions, which itself was adapted from Thalbourne (1982) glossary: “A sensory experience in which there appears to be present a person or animal (deceased or living) who is in fact out of sensory range of the experient…” (p. 110).

A principal component analysis and quartimax rotation suggested that anomalous experiences during mirror and eye-gazing sessions form three independent factors (Caputo, 2019). This same three-factor structure was confirmed via other methods (e.g., polychoric, alpha), suggesting that the perceptual anomalies derive from three distinct states of consciousness: (i) depersonalization (i.e., changes of multisensory integration on bodily-self, hence out-of-body presence); (ii) derealization (i.e., changes in sensory maps of visual processing, hence deformations in perceptions); and (iii) dissociated identity (i.e., changes with self-concept, thus apparitions of strange personalities in place of the subject’s real face reflected in the mirror). The balance among these three processing levels apparently varies among observers (Caputo, 2019).

### Lighting Levels

Illumination is an understudied topic in the relevant literature. Settings with low-light appear to be normal operating procedure in many field investigations (e.g., Houran et al., 2002b; Laythe and Owen, 2013), not to mention spiritualistic practice (e.g., Laythe et al., 2017). Moreover, the horror film genre is an obvious example of darkness being used as a “theatrical artifice” (cf. Loiselle, 2020) to reinforce conditions of spookiness or creepiness. Therefore, it is reasonable to expect that darker settings bolster the expectancy set of percipients.

Of the few studies that have directly examined lighting in relation to haunts, Terhune et al. (2007) found that overall lighting levels were not significantly different in an allegedly haunted site compared to a control site. Yet, examination of the means and standard deviations do show lower mean levels of lighting (F-stop aperture: *M* = 4.07 vs. 4.77) and much less variability (*SD* = 0.19 vs. 1.19) at the haunted location, indicating an overall lower level of lighting (albeit non-significant). A serious limitation in this study was that measurements were not made simultaneous with real-time reports of anomalous experiences. In contrast, Wiseman et al. (2003b) measured the lighting levels both inside and directly outside the test areas of the haunted South Bridge Vaults (Edinburgh, Scotland). These researchers found a significant association between the lighting outside of target areas and anomalous reports of participants, as well as with those areas with a history of ghostly reports.

Nevertheless, “lighting levels” could be the wrong attribution for these findings if the absence of light or sensory deprivation is instead the principal effect. An oft-used explanation for ghostly anomalies as a function of darkness is visual pareidolia, or the tendency to make or perceive meaningful patterns in visual noise (Myers, 2015). Nees and Phillips (2015) similarly argued that auditory pareodolia accounted for so-called “electronic voice phenomena” (EVP) and related experiences in some haunt episodes. Evidence supports this model, although it typically derives from research with patients suffering from psychosis or disorders such as dementia. For instance, Mamiya et al. (2016) standardized a short-form visual pareidolia test for use with dementia patients, which correlated positively (*r* = 0.42) with separate measures of pareidolia. This test provides a series of white noise and blurred image pictures for participants to interpret. Notably, they do not measure low lighting images, but earlier work using this procedure (Uchiyama et al., 2012) showed a significant increase in pareidolia hallucinations with dementia patients versus controls.

Unfortunately, the populations and methodologies in these studies undermine the generalizability of their findings for non-clinical samples or haunt-related contexts. More closely related to the dark bowers of a haunted location are Daniel and Mason (2015) sensory deprivation studies. These researchers placed participants (scoring either low or high on psychotic-like experience) in a sensory deprivation chamber for sound and light. Both the low and high scoring groups reported a significant increase in psychotic-like experiences, which did not appear to be a function of either suggestibility or fantasy proneness.

Overall, lighting level seems a likely contributor to experiences deemed paranormal or ghostly. However, we note that light anomalies or other curious “artifacts” captured on film or video (Lange and Houran, 1997b; Storm, 2001; Ventola, 2002; Schwartz and Creath, 2005; Laythe and Owen, 2013; Mayer, 2014) or measured outside the visible light spectrum (Joines et al., 2012) are not, strictly speaking, accounted for by pareidolia-like effects. Relative to the former, Wilson et al. (2010) demonstrated transient decreases in both infrared and visible light during environmental measurement of a single séance session of approximately 95 minutes. Further studies are needed to account for low-light pareidolia phenomena, while controlling for other environmental factors in haunt-related settings (e.g., Jawer et al., 2020).

### Air Quality

Government agencies describe the general cleanliness of the air and potentially associated health effects via the Air Quality Index (AQI: see^1^ and^2^). Five major air pollutants are regulated by the Clean Air Act in the United States: (i) ground-level ozone; (ii) particle pollution (e.g., acids, such as nitrates and sulfates); organic chemicals, metals, soil or dust particles, and allergens (e.g., fragments of pollen or mold spores); (iii) carbon monoxide; (iv) sulfur dioxide; and (v) nitrogen dioxide. For each of these, the Environmental Protection Agency has established national air quality standards and calculates the AQI to protect public health.

Of the above categories, we found only references to particle pollution and carbon monoxide in the haunt literature. For instance, humidity or water vapor is a contributor to mold growth (Environmental Protection Agency, 2017). Numerous articles have bolstered public awareness of the acute and chronic illnesses that can result from exposure to biotoxins made by molds, dinoflagellates, spirochetes, and blue-green algae (Shoemaker et al., 2005; Ackerly, 2014; Tsafrir, 2017). The ensuing symptoms sometimes parallel the psychological experiences that characterize haunts, e.g., disorientation, mood swings, temperature regulation problems, and tingling (cf. Tsafrir, 2017, para. 8).

Since many haunted locations are older structures that are prime candidates for mold or other indoor air quality problems, some authors (Clarkson University, 2015; Kane, 2015) have proposed that ghostly experiences are indicative of exposure to toxic molds. To our knowledge, this speculation has yet to be validated by research showing differences in indoor air contamination between haunted and control locations. Furthermore, the available evidence is not persuasive that haunts are even indirectly related to humidity levels (or mold growth). Terhune et al. (2007) study of a target house and control house revealed significantly higher humidity levels in the target house, but there was no statistically significant difference in humidity levels between haunted versus non-haunted rooms of the target house. There were likewise no significant differences in humidity levels (or the number of air vents) in Houran (2002) investigation of haunted and non-haunted rooms at a historic mansion.

Broadly speaking, the relevant literature has omitted the measurement of humidity. This is not to say that pertinent findings are completely absent. To be sure, “It’s not the heat, it’s the humidity” is an old adage with some empirical support. For instance, Ding et al. (2016) found that humidity significantly compounds the negative association between hot weather and mental health, demonstrating a.01% to.05% increase in negative mental health effects based on logit model prediction of heat and humidity. Still, the contribution of humidity to mental health appears to be small.

Conversely, the role of carbon monoxide has been clearly substantiated in a few reports. Perhaps most famously, Wilmer (1921) published a dramatic case study of a couple who moved into a “large, rambling, high-studded house, built around 1870, and much out of repair.” The pair soon began having anomalous experiences encompassing unusual bouts of headaches, strange sensations, feelings of listlessness, hearing phantom footsteps, and seeing mysterious figures. Their complaints closely matched the classic signs or symptoms of a haunt (Houran et al., 2019a, b), but these were eventually traced to carbon monoxide poisoning from a faulty furnace.

According to The Body Odd (2009), a much more recent case involved a woman who was found delirious and hyperventilating after seeing a ghost while taking a shower. Investigators discovered a new gas water heater had been improperly installed and thus flooded the house with carbon monoxide. Beyond those two examples (including only one citation within our literature set), the available evidence does not implicate carbon monoxide poisoning in witness reports. Telling in this respect is that Joe Nickell, a well-known skeptic and researcher of paranormal claims, stated that he has “…never encountered this scenario” (The Body Odd, 2009, para. 18).

### Temperature

Ghostly episodes can, but rarely, involve reports of an increase in temperature (Houran et al., 2019a). For example, Nickell (2001) discussed one account in which a phantom silhouette was reported by a naval captain in an unbearably hot bedroom. The most often reported temperature anomalies in haunt-type experiences, however, are so-called cold spots, i.e., a distinct perception of localized coldness (Parsons and O’Keeffe, 2006).

Williams et al. (2008) online primer for paranormal enthusiasts noted that the duration of cold spots can range from a “fleeting feeling, or they may be persistent over time” (p. 1). Parapsychologists acknowledge that these subjective temperature drops or changes at haunts may stem from the predictable physiological reaction to fear in a purportedly haunted space (O’Keeffe and Parsons, 2010). Still, there is a paucity of laboratory research to verify the hypothesized causes of temperature anomalies.

Some experimental work on related anomalous experiences includes reports of temperature drops, such as with séance room phenomena (Wiseman et al., 2003a). O’Keeffe and Parsons (2010) critically discussed one of the few studies in the last 25 years — conducted by Radin and Rebman (1996) — that tested the correlation between temperature changes in the immediate environment and participants’ mental states (albeit via the induction of an anomalous experience). The study’s protocol involved an instrumented psychomanteum chamber: a small and dimly lit room with a mirror strategically placed to induce experiences of after-death communication (Root, 2015). O’Keeffe and Parsons (2010, p. 113) noted that “some of the significant ambient temperature and physiological correlations were possible artifacts of a common downward drift in temperature” exacerbated by the floor-level placement of the computerized thermometer.

Terhune et al. (2007) extensive field study of an allegedly haunted residence found that ambient temperature was significantly colder compared to a nearby designated control house, even when possible confounds were considered (e.g., number of windows). However, there was little difference within the haunted house itself, i.e., no relationship between areas associated with anomalous experiences and temperature readings. Similarly, a series of field experiments conducted at Hampton Court Palace and the Edinburgh Vaults to examine psychological mechanisms that might underlie participants’ haunt experiences (cf. Houran et al., 2002b) also accumulated a wealth of data on environmental variables (Wiseman et al., 2003b). The researchers found no significant relationship between temperature and the number of anomalous experiences that research participants reported (Wiseman et al., 2003b).

### Infrasound

Leventhall et al. (2003) defined infrasound as audio frequency energy that falls below the range of normal hearing, typically 20Hz. It can be characterized simplistically as a hum you cannot hear. Persinger (1974, 2014) noted the prevalence of both ambient infrasound within the environment (via natural phenomena such as geomagnetic activity, wind, etc.) and man-made infrasound (e.g., aircraft, large machinery, air movement in duct systems). The vibroacoustic effect of a wide spectrum of low-frequency sound (typically 20–160 Hz) within a paranormal context has been argued from a physics perspective in two key articles (Vinokur, 2005, 2016). In these papers, Vinokur described how naturally occurring vibroacoustic phenomena can produce poltergeist-type effects (rattling windows, whispering galleries, etc.).

We note that the proposed role of infrasound in haunt-type experiences dates a decade earlier to the research of Tandy and Lawrence (1998). They posited a causal link between infrasound and apparitional experiences specifically noting that infrasound around 19 Hz appeared to cause visual effects derived from eyeball-vibration that might be interpreted as a ghostly sighting.

This basic hypothesis was tested a few years later in a study of ambient infrasound in a reputedly haunted 14th century cellar beneath a tourist information centre in Coventry (Tandy, 2002). Parsons (2012), however, conducted a series of infrasound measurements at the same venue in 2006. His findings did not support those of Tandy but instead implicated a “broad range of frequencies exceeding 30 dBS between 20 Hz and 2 HZ, with a peak at 44 dBS at 5.7 Hz” (Parsons, 2012, p. 165). Authorities have further voiced two major concerns about Tandy’s work (Tandy and Lawrence, 1998; Tandy, 2002): first, the lack of detail provided about the infrasound measurements themselves (weighting filter unspecified, room dimensions not taken into account, etc., Parsons et al., 2008; Parsons, 2012); and second, the lack of evidence demonstrating the physiological effects of such weak infrasound levels (Braithwaite and Townsend, 2006).

These criticisms challenge the relevance of infrasound (specifically around 19 Hz) to haunt-type experiences. Field studies of haunts across the United Kingdom led by Parsons et al. (2008), Parsons (2012), Parsons and Cooper (2015), on the other hand, concluded that high ambient levels of infrasound (at varying frequencies) did contribute to witness accounts. That said, these findings did not support Tandy and Lawrence (1998) hypothesis that infrasound near 19Hz induces visual disturbances that are intepreted as apparitional experiences. Nevertheless, similarities exist between the physical and psychological effects of infrasound documented in the lab and those reported anecdotally by witnesses in haunt cases (O’Keeffe and Parsons, 2010; Parsons, 2012). Participants in recent studies of pure infrasound tones at high sound-pressure levels have reported effects such as headaches, ear pressure, tiredness, change in heart rate, disorientation, and complications arising from the impact on the inner ear (Chen and Hanmin, 2004; Hansen, 2007).

Furthermore, Tandy and Lawrence (1998) original hypothesis has been re-examined in a series of unusual and highly public studies conducted since 2001. Music concerts that incorporated man-made infrasound were held at a venue in Liverpool and again at the Royal Festival Hall in London (Arenda and Thackara, 2003). In a pre-specified number of pieces during the concert, infrasound was added and the audience’s emotional response to the music correspondingly measured. Although the environment and social context may have played a role in the way the audience reacted to the music, counter-balancing of the infrasound presence over two performances controlled some of this influence. In addition to questionnaire-based data, free response sections provided a rich source for more qualitative information. Reactions ranged from low-arousal reports like “calm” and “sleepy” to more active states such as “aroused” and “excited.” These accounts reflected experiences that varied in intensity from slight agitation or light-headedness to the more noticeable, e.g., increased heartbeat, facial tingle, and a marked sense of presence (Arenda and Thackara, 2003).

A similar approach to infrasound generating and testing was used in two further public performances in 2006 and 2010, whereby the focus was on infrasound at 18.9Hz at a sound pressure level exceeding 90DBs (Forsyth and Pollard, 2019). Reactions noted by audience members included distinct physical discomfort and anxiety, yet it is unclear whether these responses were due to the generated infrasound, the ambient infrasound already present, or other environmental variables that factored into the performance (e.g., subliminal suggestion) (Parsons, 2012).

A novel test of Tandy’s hypothesis involved the construction of a “completely empty, white, and circular room” that became “haunted” through the systematic variation of two key factors: electromagnetic fields and infrasound (French et al., 2009, p. 621). In this so-called Haunt Project, participants were informed in advance that they might be exposed to varying EMFs, infrasound, both or neither, and that they might experience mildly unusual sensations as a result (p. 624). The participants spent nearly an hour wandering around the specially constructed room and were asked to record their impressions and experiences. Participants reported many unusual or anomalous perceptions, but the frequency was unrelated to the environmental manipulations. The researchers therefore proposed that expectancy or suggestion effects accounted for the participants’ experiences (French et al., 2009).

We should qualify that the apparent suggestion effects in this experiment might not have been independent of some confounding physical influences. Particularly, French et al. (2009) stated that “Informal pilot testing had suggested that dim illumination and a cool temperature would be the most suitable conditions for this study, insofar as they are the conditions typically associated with reputedly haunted locations” (p. 621). Furthermore, Parsons and Cooper (2015) were critical of the general results, raising concerns about the production of infrasound (combining two sine waves of 18.9Hz and 22.3Hz), the lack of detail regarding the sound recording equipment, and the absence of ambient infrasound data.

### Electromagnetic Fields (EMFs)

If the public associates anything that is “scientific” with haunt-type experiences, it is the apparent role of EMFs (Houran, 2017; Massullo, 2017). Indeed, it can be argued that the majority of findings from fieldwork studies of haunts relate to EMF effects (Houran and Lange, 1998). Interested readers are encouraged to consult important discussions of this topic for insight into the technicalities involved and corresponding debates concerning the issues of measurement and interpretation of research findings (see Persinger and Koren, 2001b; Williams et al., 2007; Braithwaite, 2008, 2010, 2011; Parsons, 2015).

As basic background, geomagnetic fields (GMF) are DC fields that are largely generated through the fluid motion of the Earth’s molten iron core (Buffet, 2000). Although the GMF of the Earth averages around 500-milliGauss (MG), and is typically less than 10 Hz, several variables can produce notable changes in GMF strength around the globe. These include seismic activity along fault zones (Persinger, 1974, 1985), electrical activity during thunderstorms, and large amounts of magnetic or electrically conductive minerals present in the geology of a given area. In addition, increases in cosmic radiation – e.g., from sunspots, solar flares, or similar phenomena –can sometimes greatly change GMF strength and lead to geomagnetic storms as this radiation interacts with the boundary of the GMF in the upper atmosphere (Lyon, 2000).

In contrast, electromagnetic fields are AC fields that are usually produced artificially by electrical power currents, though in some instances, EM fields are produced naturally by geophysical sources. For instance, electricity can be produced through seismic pressure acting on conductive rock along fault zones (Persinger, 1985, 1987), as well as by very low frequency atmospherics, i.e., electromagnetic pulses produced from electrical discharges after a lightning strike averaging around 0.6-MG (Schienle et al., 1998).

GMF and EMF are both phenomena associated with the electromagnetic spectrum at its slowest frequencies. Whereas GMF resides in the single-digits frequencies, EMF is typically shorthand for mains frequency (i.e. power lines) magnetic fields produced at either 60 or 50Hz, depending on your country of origin. Nowhere are the issues of technology and measurement more problematic than with EMFs (Laythe, 2015; Laythe et al., 2017), particularly in the stark contrast between laboratory designs and field measurements. Studies examining EMF-relationships have been published sporadically for years, but considerable variations in methods and ontological assumptions have made it difficult to compare and contrast study outcomes and implications.

Foremost among these issues are incorrect assumptions about EMF behavior in natural settings. Laythe et al. (2017) have emphasized that EMFs are subject to rapidly declining strength as a function of distance, which implies an exponential decay rate (Tipler, 1987; Thidé, 2004). Thus, power lines or electrical towers have been erroneously blamed for EMF findings when actually these structures can be relatively close and not affect the EMF levels of nearby environments. Similarly, most of the magnetic force of artificially produced EMF is diminished as a function of radio and broadcasting data (Thidé, 2004). Further, triangulation is rarely used with EMF in the field, which makes detection of the precise source of EMFs nearly impossible. Finally, the technology of EMF meters is receptive, meaning these have a limited capacity to detect EMF fields (which decay quickly). Readings can consequently be significantly altered by moving an EMF meter by two or three feet (Laythe et al., 2017).

These statements are neither meant to imply that EMFs do not generally affect environmental systems, nor that geophysical effects do not necessarily influence “haunted” environments. We conclude only that the methods of EMF data collection in the field have inhibited effective cross-study comparisons. For example, some evidence suggests that sleep disturbances, mood shifts, and increases in anxiety can coincide with changes in geomagnetic field activity (Persinger, 1987). Other studies suggest that people with particularly sensitive temporal lobes (a condition that may be caused by temporal lobe epilepsy or brain injury), may be more susceptible to changes in GMF activity (Fuller et al., 1995; Persinger, 2001; Persinger and Koren, 2001b, pp. 183–184).

Correlational research suggests that geomagnetic activity may be stronger on days when people report bereavement hallucinations (i.e., apparitions of people who have recently died) (Persinger, 1988; Persinger and Schaut, 1988). Strong geomagnetic fields, around 200-MG or more above the average for the Earth’s GMF, have been documented at reputed haunts (Roll and Persinger, 2001). It is important to note, however, that all these studies assume that within the mix of EMF magnitude (i.e., field strength), a persistent frequency exists that corresponds to the precise frequency needed to produce a sensed presence or related hallucinatory-type phenomena.

Some experimental evidence also raises concern over the potential effects of EMF exposure on mental health (O’Connor, 1993; Paneth, 1993). For example, two studies have observed possible changes in brain wave activity on an electroencephalogram (EEG) following two-second exposure to EMFs as strong as 780-MG (von Klitzing, 1991; Bell et al., 1992). Persinger et al. (1997) found changes in brain waves when lower strength magnetic fields (10-MG) were applied over several minutes, and these changes persisted a short time after the magnetic stimulation ceased.

A review of experimental studies also suggests that brain chemistry and hormone levels may sometimes change in response to EMF exposure (Reiter, 1993). Some data also suggest that EMF exposure can also affect sleep (Sher, 2000), which might contribute to haunt experiences that occur during sleeping hours. Gangi and Johansson (2000) even proposed that EMF exposure can cause certain skin cells to release inflammatory substances that can cause itching and other skin sensations. Such physiological effects might relate to unusual somatic complaints reported in some haunt experiences (Houran et al., 2002a; Houran et al., 2019a).

EMF strength in buildings typically averages between 0.2- to 2-MG. Several field investigations of haunts have measured EMFs appreciably above this average (e.g., Roll et al., 1996; Persinger et al., 2001; Roll and Persinger, 2001, pp. 154–163; Wiseman et al., 2002). In the laboratory, Persinger et al. (2000) studied the experiences of a man who had reported haunt phenomena in his home. When they applied a 10-MG EMF to his brain, the man reported experiencing brief rushes of fear and various odd sensations. This was followed by his perceiving a visual image that seemed to resemble the apparition he had remembered previously. Changes in brain wave activity were also measured via EEG in conjunction with his anomalous perception (for discussions of this and related work, see Persinger, 2001; Persinger and Koren, 2001a, b).

Laboratory research also shows that anomalous impressions can be artificially induced by stimulating the brain with temporally complex, weak-intensity magnetic fields (Cook and Persinger, 2001; Persinger, 2001, Persinger, 2003; Persinger et al., 2001; for reviews see Persinger and Koren, 2001a, b). According to Persinger, anomalous perceptions are caused by temporally complex magnetic fields that induce partial micro-seizures (paroxysmal events) in temporal-lobe regions and the deep sub-cortical structures they house, such as the hippocampus and amygdala (Persinger and Koren, 2001b).

Essentially, Persinger proposed that such micro-seizures can cascade through the neural landscape and, with enough intensity, affect the individual’s thoughts, images, memories, and feelings, so that hallucinations, and anomalous perceptions result (Persinger and Healey, 2002). His clear and testable prediction is that magnetic fields present at some reputed haunts can induce reports of sensed presences or other ghostly experiences (Persinger et al., 2001; Persinger and Koren, 2001b; Roll and Persinger, 2001). However, this “Persinger effect” (i.e., EMF-induced hallucinations) as a comprehensive explanation for haunted houses is insufficient for several reasons.

First, it does not consider the low probability that all haunts exist at environments where a very specific and precisely patterned EMF wave can affect temporal lobe functioning. Braithwaite (2008) haunt investigations, for example, identified only two of approximately 50 sites with magnetic fields that were temporally complex. This incidence rate (4%) might be better described as “coincidental” than “rare.” Similarly, Laythe and Owen (2013) found highly varied EMF and GMF readings in a non-powered, electrical environment. This suggests that ostensibly anomalous EMF/GMF is not stable over time. Thus, it remains to be seen whether the waveforms measured and detailed in these studies have any implications for human experience, even in contextually and experientially rich settings.

Also notable is the fact that one particular study, while failing to replicate the Persinger effect, implicated the role of suggestion and prior belief (Granqvist et al., 2005). Persinger and Koren (2005) subsequently criticized Granqvist and colleagues by claiming that the fields used may not have been appropriate for eliciting a neurological response, possibly due to alterations in the temporal characteristics of the waveforms (for a reply, see Larsson et al., 2005). Persinger’s argument implies that a high degree of temporal specificity is required to elicit the hypothesized effects. His earlier studies, employing an apparatus known as the “God Helmet” (where magnetic coils are strategically temporally placed) were, in fact, partially replicated by a team whose production of 10mG magnetic fields in the helmet resulted in participants reporting anomalous perceptions, including sensed presences (Tinoco and Ortiz, 2014).

Persinger’s ideas presumably have limited applicability to most haunt sites since the requisite temporal complexity is unlikely to occur. We note, furthermore, that many studies have found no such effects. These include several field investigations (Maher, 2000; Wiseman et al., 2003b) and laboratory experiments (French et al., 2009). Williams (2015) duly noted a lack of a historical relationship between reported haunts and manufactured EMFs. Cornell (2002) similarly pointed out that haunt accounts “…were widely reported long before the development and use of man-made electromagnetic utilities” (p. 388).

Still, several haunt investigations have documented EMF effects and found that the absolute strength or intensity of the magnetic fields might not be as important as their fluctuation over time. These studies have sought to quantify the magnetic fields at reputed haunts and compare them to appropriate baselines (Wiseman et al., 2002, 2003b; Braithwaite, 2004, 2008; Braithwaite et al., 2004; Braithwaite and Townsend, 2005; Terhune et al., 2007; Laythe and Owen, 2013). In at least two field investigations by William G. Roll (reported in Roll and Persinger, 2001), the strength of the magnetic fields was found to be either gradually increasing or decreasing as one moved from one side of the haunted site to the other. During a study of haunt reports at historic Hampton Court Palace, Wiseman et al. (2002, 2003b) noted that changes in the magnetic fields in areas of the palace associated with anomalous experiences differed significantly from the EMF changes in control areas in which no such experiences were reported. Variance of the magnetic field correlated with the number of unusual experiences reported.

Braithwaite and colleagues examined a specific bedroom at the historic Muncaster Castle on multiple occasions (Braithwaite, 2004; Braithwaite et al., 2004). Witnesses sleeping in the room had reported hearing voices at night that resembled children crying. Braithwaite’s group took measurements around the head of the bed and then compared them to measurements taken toward the center of the room where the mysterious voices apparently originated. Notable changes in magnetic field strength were observed over this short distance of a few meters. Similarly, Terhune et al. (2007) found noteworthy differences when comparing the magnetic field changes in areas where haunt phenomena were reported with control areas that had no reported phenomena.

Other researchers have suggested that the potential influence of magnetic fields might be greater if they exist within built environments that are ‘spooky’ (e.g., feature gothic architecture, dim lighting, or vintage paintings and furniture: Lange and Houran, 1997a; Houran, 2002; Braithwaite and Townsend, 2005; Braithwaite, 2008; Ralphs, 2012). It might be possible that such contextual variables work together with magnetic fields to stimulate expectation as well as neural arousal.

Recent research further complicates the EMF-haunted house relationship. Wilson et al. (2010) found changes in EMF during a séance session where light anomalies and rappings sounds occurred. Additionally, two detailed studies appear to show significant real-time associations between EMFs and clearly physical (vs. imagined) anomalies (Laythe and Owen, 2013; Laythe and Houran, 2019). In these latter studies, anomalous phenomena captured in audio or video recordings were shown to correlate with significant micro-expansion or -suppression of the area’s EMF field during the time period of the documented anomalous event.

Also, hourly correlations of EMF/GMF meters in the Laythe and Owen (2013) study varied wildly on an hourly basis in a location that was approximately a half-mile from any confounding electrical sources. Further investigation by Laythe et al. (2017) in a laboratory séance setting found significant variability of EMF and GMF across sessions, and EMF-spikes were significantly associated with participants’ anomalous experiences. This suggests that micro-expansion or micro-contraction of EMF may be a significant factor in haunt-type experiences.

Laythe’s three studies above appear to challenge conventional physical explanations for the observed EMF effects. His work further undermines a hallucinatory-EMF model as the sole explanation for haunt-type experiences. Both Laythe and Owen (2013) and Laythe and Houran (2019) recorded objective anomalies (i.e., tangible and measurable) in tandem with significant EMF fluctuations. Given that EMF manifests as either a vector (a focused wave with direction), or a general field with a source of origin, neither study could account for any EMF source that could theoretically create localized variability in the EMF field. Although these micro EMF expansion and suppression effects have been conceptually replicated three times in different environments, they require further independent validation.

## Discussion

Examination of the identified literature showed that the methodological recommendations put forward by previous authorities (i.e., Persinger and Koren, 2001b; Radin, 2001; Roll and Persinger, 2001; Schmeidler, 2001) had not been consistently adopted by investigators. That is, few research designs considered either a range of environmental variables simultaneously, compared results from haunted locations to suitable control conditions, or applied extensive or competitive hypothesis testing using collaborations between investigators with ideological differences.

Researchers instead tended to pinpoint several conventional factors that can theoretically impact, though perhaps subtly or unwittingly, the psychological experience of natural and built environments. Yet, this target literature offered neither abundantly clear, nor persuasive evidence for most of these ostensible unconscious or conscious stimulants as a robust model for haunted houses. Specifically, it appears that the hypothetical influence of environmental variables touted by some authors (e.g., Houran, 1997; Tandy and Lawrence, 1998; Vinokur, 2005, 2016; Alexander and Muzzillo, 2010/2014; McAndrew, 2015, 2020) does not consistently match their observed influence.

We conclude therefore that an exclusively or chiefly environmental model — i.e., relying on discrete embedded cues, air quality, temperature, infrasound, lighting-levels, or electromagnetic fields — is presently insufficient as a general explanation for what imprints certain locations or settings with a haunted persona (or creepiness) or serves as the predominant source of anomalous experiences in these contexts. That said, it is highly questionable that evolutionary-environmental perspectives on ghostly episodes have been adequately explored or tested, despite the long legacy of fieldwork studies and instrumentation in haunt-related research (for overviews, see e.g., Osis, 1982; Houran and Lange, 1998; Braithwaite, 2006; Parsons and O’Keeffe, 2008; Bebergal, 2018; Radford, 2018).

Moreover, we continue to anticipate slow advancements in understanding “haunted houses” given their taboo standing within many academic circles and publications. Amateur enthusiasts of the paranormal tend neither to be professionally trained nor scientifically oriented (Potts, 2004; Hill, 2017; Hill et al., 2019; Eaton, 2015), yet they dominate the popular view of haunt investigation and accordingly taint a fascinating and valid subject. This situation is unfortunate, because empirical study in this domain transcends parapsychology to be potentially instructive for exploring or refining important issues across the social and biomedical sciences. These include sensory processing sensitivity and perceptual biases (van Elk, 2015; Partos et al., 2016; Greven et al., 2019), sick building syndrome (Shoemaker and House, 2006), mass (contagious) psychogenic illness (Chen et al., 2003), embodied-cognition (Goldhagen, 2017), the neurobiology and physiology of emotion (Jawer and Micozzi, 2009), place identity and attachment (Donohoe, 2014; Seamon, 2014), the nature of creepiness (McAndrew and Koehnke, 2016; McAndrew, 2020), extraordinary architectural experiences (Bermudez, 2009; Bermudez and Ro, 2018), and the psychology of sacred or enchanted spaces (Lidov, 2006; Holloway, 2010).

Accordingly, scientific efforts to describe haunted houses and related phenomena in environmental terms should address several issues. First, our literature review revealed a dearth of detailed and quality research in this area. Future studies must therefore strive to measure discrete physical factors more consistently, comprehensively, and precisely. Fieldwork should include experts in architectural design, engineering, environmental sciences, and physics. Such specialists in environmental and architectural phenomenology could be invaluable in developing or implementing new research designs. Furthermore, investigations must consider individual differences in the attentional, emotional, and perceptual thresholds of experients versus non-experients in haunt-type cases (Kumar and Pekala, 2001; Lange and Houran, 2001a, b; Houran et al., 2002a; Jawer, 2006; Romer, 2013; Laythe et al., 2018; Parra, 2018; Ventola et al., 2019). It could be that the types of physical variables reviewed here are germane to a subset of witness reports grounded in hypervigilance or heightened sensitivities to these conventional stimuli.

“Gestalt influences” are additional factors whose role in this domain have yet to be understood. These are ambient, structural, or contextual variables that have the capacity to influences a person’s perceptions, feelings, and impressions of specific spaces and settings. Jawer et al. (2020) discussed several examples relevant to haunt-type experiences, including: (i) affordance, (ii) atmosphere, (iii) ambiguity and threat anticipatory processes, (iv) immersion and presence, (v) legibility, and (vi) percipient memory and associations (e.g., transgenerational, transpersonal, and archetypal memories: Jung, 1979; Caputo, 2017) that can be involved in apparitional/spiritual phenomena and are specifically encoded or contextually re-encoded through haunted, enchanted, and sacred places. These effects might involve, but are not limited to, the discrete physical factors proposed as stimulants of anomalous experiences. Gestalt influences instead speak to the larger concept of systems theory (i.e., environment-person bidirectional or enactive processes) (Jelić et al., 2016; Goldhagen, 2017).

This holistic view identifies psychosocial elements as important contributors to the onset or structure of personal experiences, which agrees with conclusions from our broad sociocultural analyses of ghost narratives (Hill et al., 2018, 2019; Drinkwater et al., 2019; Houran et al., 2020). To be sure, considerable evidence implicates attitudinal, normative, and situational influences in the phenomenology of witness accounts (Houran, 2002; Houran et al., 2002b, 2019a; Wiseman et al., 2002, 2003b; French et al., 2009; Drinkwater et al., 2013, 2017; Dagnall et al., 2015a; Laythe et al., 2018; Pharino et al., 2018; Langston and Hubbard, 2019).

Such findings underscore that physical variables might not be the primary culprits in most haunt cases. For instance, Aulet and Vidal (2018) stated that “Sacred spaces are complex realities whose internal dynamics must be studied from a multidisciplinary and transversal perspective that draws on anthropology, sociology, theology, philosophy, tourism, culture and more” (p. 255). Likewise, haunted houses could be variants of enchanted or sacred spaces (Jawer et al., 2020) and thus may have eluded definitive explanation for millennia due to roots in complex interactions (or dynamical systems; e.g., Lange and Houran, 2000, Lange and Houran, 2001b) among certain physical variables, sociocultural influences, situational context, and interpersonal dynamics — all of which shape the character of spaces and settings, as well as define how experiencers are ultimately situated inside them.

In closing, we would be remiss not to mention an environmental model for haunts taken to the extreme. This is the possibility that human consciousness, and indeed all that we experience as reality, derives from a sophisticated hologram or computer program, as depicted in the sci-fi film franchise, *The Matrix*. Academics refer to this as the “simulation hypothesis”, and if valid it implies that ghosts are quite literally in the machine. That is, the anomalies that characterize haunt-type episodes might represent glitches in the software or hardware that produces or operates the simulation.

The idea that ghostly phenomena can be interpreted in informational terms parallels some thinking in parapsychology (Radin, 2018). More pointedly, Merali (2013) outlined conceptual and empirical arguments consistent with the simulation hypothesis, and “Is the Universe a Simulation?” was even the central topic of the 2016 Isaac Asimov Memorial Debate at the American Museum of Natural History (cf. Moskowitz, 2016). This intriguing notion, like that of ghosts and parapsychological agencies, might be a stretch and eventually prove incorrect. Nevertheless, asking these types of questions underscores the fundamental need that human beings apparently have to explore and understand all facets of their holistic environments. We sympathize with paranormal experients in this regard because academia does not yet have a convincing, comprehensive, and scientific explanation for haunted houses — and without dedicated and inclusive research it never will.

## Author Contributions

ND performed the theoretical focus, design, provided theoretical background and draft feedback. KD performed the theoretical focus and provided theoretical background and draft feedback. CO’K commented on drafts and provided theoretical background. AV, BL, MJ, BM, and GC provided theoretical background and draft feedback. JH provided theoretical focus, design, theoretical background, and draft feedback.

## Conflict of Interest

The authors declare that the research was conducted in the absence of any commercial or financial relationships that could be construed as a potential conflict of interest.

## Footnotes

1 https://airnow.gov

2 https://bit.ly/306pDEc
